# The interaction of seasonal forcing and immunity and the resonance dynamics of malaria

**DOI:** 10.1098/rsif.2009.0178

**Published:** 2009-07-01

**Authors:** Dylan Z. Childs, Michael Boots

**Affiliations:** Department of Animal and Plant Sciences, University of Sheffield, Alfred Denny Building, Western Bank, Sheffield S10 2TN, UK

**Keywords:** seasonality, immunity, resonance, malaria, multi-annual cycles, resonance approach

## Abstract

Theory has emphasized the importance of both intrinsic factors such as host immunity and extrinsic drivers such as climate in determining disease dynamics. In particular, seasonality may lead to multi-annual cycles in prevalence, but the likelihood of this depends on the role of acquired immunity. Some diseases including malaria have immunity that falls between the classic susceptible–infectious–removed and susceptible–infectious–susceptible models. Here, we investigate the general conditions promoting the subharmonic resonance behaviour that may lead to multi-annual cycles in a general malaria dynamical model. Utilizing two complementary approaches to bifurcation analyses, we show that resonance is promoted by processes shortening the length of the infectious period and that subharmonic cycles are favoured in situations with strong seasonality in transmission but at intermediate levels of endemicity. We discuss the implications of our results for understanding prevalence patterns in long-term malaria datasets from Kenya that show multi-annual cycles and one from Thailand that does not and discuss the possible implications of treatment.

## 1. Introduction

Understanding the mechanisms that underpin the dynamics of important human diseases remains a key challenge for epidemiologists. Perhaps the most important intrinsic driver of infectious disease epidemiology is the immune interaction (Anderson & May 1991; Grassly *et al*. 2005). It is well known that the unforced susceptible–infectious–removed (SIR) model can exhibit damped oscillations in the presence of host immunity (recovery), because susceptible numbers take time to build up following an epidemic. In the face of weak environmental variation or demographic stochasticity, resonance ensures that such fluctuations are sustained at a characteristic natural period (*p*_0_). In contrast, models of the susceptible–infectious–susceptible (SIS) variety, without acquired immunity, are much less prone to oscillatory dynamics under comparable conditions. However, many important diseases have more complex immune interactions than the two extremes of SIR and SIS. For example, a key characteristic of malaria is that substantial immunity is achieved after multiple infections and that this immunity may be maintained in the face of repeated exposure to infection, a phenomenon occasionally referred to as immune boosting (Struik & Riley 2004). Malaria therefore lies somewhere between the two extremes of the SIR and SIS, because boosting ensures that the flow of susceptible individuals back from the recovered class depends upon the level of disease transmission.

Recently, there has been a growing interest in the development of predictive modelling tools that are able to link climate determinants of vector abundance to malaria transmission and ultimately to disease burden (Rogers *et al*. 2002). In order to be effective, these early-warning systems may need to incorporate intrinsic drivers of epidemiological dynamics such as host immunity, in addition to extrinsic drivers such as climate. Recent work on measles (Earn *et al*. 2000; Grenfell *et al*. 2002), syphilis (Grassly *et al*. 2005) and cholera (Koelle & Pascual 2004; Koelle *et al*. 2005) has emphasized the importance of these intrinsic drivers and further highlighted the important role of mechanistic models for understanding the epidemiology of infectious disease. Malaria caused by *Plasmodium falciparum* is one of the largest causes of morbidity and mortality in the developing world and creates an enormous barrier to economic and social development (Sachs & Malaney 2002). A clear understanding of the underlying mechanisms that drive malaria epidemiology is therefore vital if we are to effectively predict and manage outbreaks and reduce levels of infection. Our aim is to use the mechanistic approach to gain a better general understanding of the drivers of malaria dynamics.

One factor limiting our understanding of malaria is a paucity of long-term detailed time-series data. However, an analysis of the periodicity of malaria hospital admission data from the Kericho region of Kenya found clear evidence for a multi-annual cycle in prevalence, with a mean of approximately 3 years (Hay *et al*. 2000, 2001). Most of the variance in the contemporaneous climate data was associated with cycles of a year or less, suggesting that intrinsic rather than extrinsic factors might be important in generating the observed cycles. Another analysis of these data again suggested that longer term multi-annual cycles were likely to be driven by intrinsic disease dynamics (Pascual *et al*. 2008). In a similar analysis of malaria prevalence in northern Thailand, we found no clear evidence for super-annual cycles (Childs *et al*. 2006). An improved understanding of the relationship between extrinsic and intrinsic drivers of malaria and their concomitant effect on disease dynamics is clearly needed in order to better understand these different patterns in different countries.

A number of factors affecting malaria transmission, including vector density and the development time of the parasite, depend upon key climate variables such as rainfall and temperature (Bayoh & Lindsay 2003, 2004). In turn, these climate variables may exhibit a strong annual component of variation, leading to the seasonal forcing of malaria transmission. Analysis of the forced SIR class of models (and S(E)IR (E—exposed) where there is an exposed, latent class) has demonstrated that periodic forcing can interact with nonlinearities in the model to generate novel subharmonic oscillations, with periods at an integer multiple of the forcing period (Dietz 1976; Aron & Schwartz 1984). In addition, subharmonic resonances of different orders may coexist simultaneously, resulting in a system with multiple stable states. This mechanism has been invoked to explain the range of complex dynamics observed in childhood diseases such as measles (Earn *et al*. 2000; Greenman *et al*. 2004). In S(E)IR models of childhood diseases, subharmonic resonances are excited under conditions of relatively low amplitude forcing and short infectious periods. In malaria, there is the potential for transmission to vary enormously over the course of a year and the infectious period may last for a number of weeks. The dynamical consequences of the interaction between strong seasonal forcing, longer infectious periods and boosted host immunity have not been investigated.

Here we develop a mechanistic model that examines how differences in key epidemiological parameters may contribute to observed differences in the dynamics of malaria infection. The model is based upon the classic Ross–Macdonald framework (Ross 1911; Macdonald 1957) and is motivated by the extensions developed by Aron (1988) and Aron & May (1982). In order to simplify the analysis, we assume that complete immunity is achieved after first infection, but that the maintenance of immunity is facilitated by higher overall transmission. We also assume that the period of immunity following recovery or boosting is exponentially distributed (i.e. the loss rate of immunity is constant) and that boosting events proceed independently of one another. The resultant model is parametrized in terms of 11 terms describing the disease epidemiology and the life history of the vector and host. However, over a biologically realistic range of values for the human mortality rate, the dynamical behaviour of the model is determined by four key parameters: the forcing amplitude *δ*, the recovery rate *γ*, the immunity loss rate *ρ* and a transmission term, the mean vectorial capacity *C*_0_. This last term is a product of several remaining parameters, such that a comparable proportional change in any one of these has the same impact on the model behaviour. We begin by exploring the general properties of the model, focusing on the conditions promoting subharmonic resonance. The model is then used to suggest a possible explanation for the different temporal patterns of malaria prevalence observed in Thailand and Kenya.

## 2. The model

The host population is divided into the proportion *x* of susceptible individuals, the proportion *y* of infected individuals and the proportion *z*_*i*_ of individuals currently immune with *i* concurrent boosting events. This gives rise to the following system of ordinary differential equations:
2.1
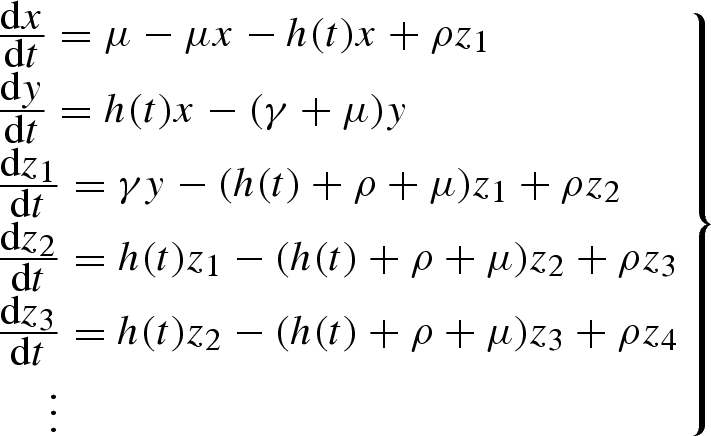

This is subject to the usual constraint

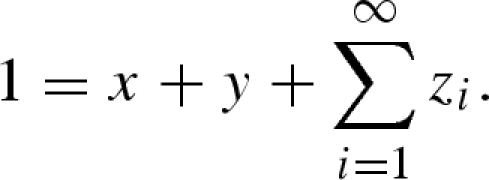

The parameter *μ* is the human death rate, *ρ* is the immune function loss rate, *γ* is the recovery rate and *h*(*t*) is the inoculation rate. The term *h*(*t*) is derived by assuming that the vector dynamics are fast compared with those of the host (see Koella & Anita (2003) for an example of the derivation). This allows the inoculation rate to be expressed as
2.2
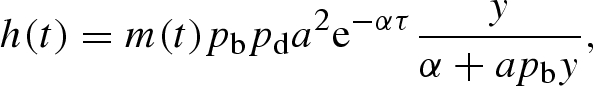

where *m*(*t*) is the number of vectors per host, *a* is the biting rate of the vector, *α* is the vector death rate, *τ* is the incubation period of the parasite, *p*_b_ is the proportion of bites on an infected host leading to infection of the vector and *p*_d_ is the proportion of bites from an infected vector leading to infection or immune boosting of the host population. Seasonality is introduced by allowing the vector–host ratio *m*(*t*) to vary sinusoidally over the year such that 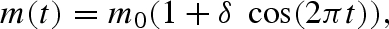 where *m*_0_ is the mean vector–host ratio and *δ* is the forcing amplitude. The model is further simplified by noting that the potential inoculation rate is approximately linear in *y* when *α* ≫ *ap*_b_*y*, such that
2.3
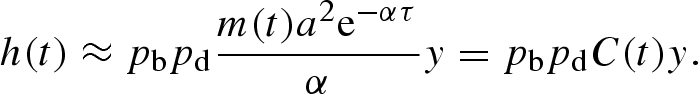

This linear approximation to the inoculation rate works well over the range of biologically plausible parameter combinations. The term *C*(*t*) is an epidemiological index called the vectorial capacity. It describes the rate at which a single case of malaria generates new bites from infected mosquitoes when *p*_b_ = *p*_d_ = 1. We refer to the mean value of this parameter as the mean vectorial capacity, *C*_0_. Consequently, it is used throughout the analysis to restrict the number of parameter combinations that need to be explored. The basic reproductive rate (*R*_0_) of malaria in this model is given by the standard expression 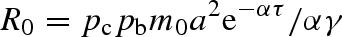.

In order to numerically integrate the model and perform the usual local stability analysis calculations, we need to close the system of equations given above. This is achieved by noting that in the absence of forcing, the non-trivial equilibrium ratio of densities in successive immune classes (*σ*) is constant, i.e. *σ* = *z*_*i*__+1_/*z*_*i*_ = *z*_*i*__+2_/*z*_*i*__+1_ = *z*_*i*__+3_/*z*_*i*__+2_ ⋯, for *i* in [1, ∞]. We conjecture that even in the presence of large periodic perturbations this ratio is approximately constant as *i*→∞. Numerical simulation of the forced system with a large number of immune classes (e.g. 1000) supports this assumption for a wide range of parameters: although there are periodic fluctuations in the magnitude of these ratios, the amplitude of these fluctuations declines geometrically along the sequence of immune stages. Therefore, we can capture the dynamics of the system by explicitly modelling an arbitrary number of immune classes (*N*), while implicitly modelling the remaining classes by solving for *σ*(*t*) under the assumption that *σ*(*t*) is identical for all classes. This gives rise to the following equations for the dynamics of the immune classes:
2.4
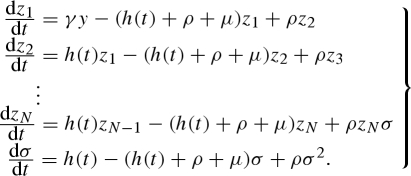

Under a wide range of parameter values, time series from periodically forced populations with 1000 immune classes are indistinguishable from those using only a small number of immune classes, even when the *z*_*i*__+1_/*z*_*i*_ are close to 1. Therefore, numerical simulations and stability analysis are conducted with 12 immune classes throughout this study.

## 3. Bifurcation analysis

**Figure 1.**
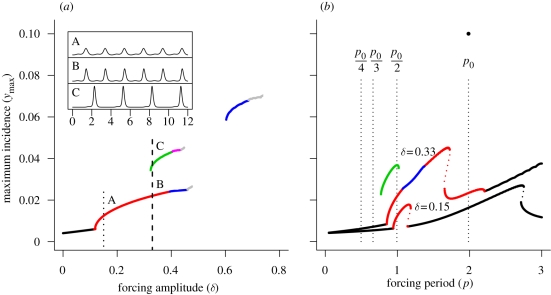
(*a*) Bifurcation diagram showing the resonant states of the baseline model under annual forcing in response to the forcing amplitude, *δ*. Dotted line, *δ* = 0.15; dashed line, *δ* = 0.33. The parameters of the baseline model are: human death rate, *μ* = 0.02 yr^−1^; immunity loss rate, *ρ* = 0.5 yr^−1^ (average immune period following recovery or immune boosting, 2 years); recovery rate, *γ* = 17.3 yr^−1^ (average infectious period, 3 weeks); mean number of vectors per host, *m*_0_ = 25; parasite incubation period, *τ* = 0.03 yr; proportion of bites on an infected host leading to infection of the vector, *p*_b_ = 0.5; the proportion of bites from an infected vector leading to infection or immune boosting of the host population, *p*_d_ = 0.5. The composite parameter summarizing transmission and boosting (vectorial capacity) is *C*_0_ = 573 yr^−1^. *Inset*: (A) period 2 oscillation, *δ* = 0.15; (B) period 2 oscillation, *δ* = 0.33; (C) period 3 oscillation, *δ* = 0.33. (*b*) Resonance diagrams for the baseline model when *δ* = 0.15 and *δ* = 0.33. The vertical dotted lines locate submultiples of the natural period. Black line, *p*_S_ = 1; red line, *p*_S_ = 2; green line, *p*_S_ = 3; blue line, *p*_S_ = 4; pink line, *p*_S_ = 6; grey line, *p*_S_ ≥ 8.

In the absence of seasonal forcing, the malaria model is stable inside the range of biologically plausible parameter values. The resonance structure of the seasonally forced model can be explored using bifurcation analysis; numerical simulation of the model is used to determine the availability of different resonance modes (multi-annual cycles with different periods) as parameters vary. When the forcing amplitude is sufficiently small, the system responds with oscillations of the same period as the external forcing. We refer to this as the base mode. Stable multi-annual cycles are only observed as the strength of forcing increases. This is illustrated in the bifurcation diagram in figure 1*a*, where the maximum prevalence in the infected population, *y*_max_, is plotted against the forcing amplitude parameter, *δ*. Multi-annual cycles of period 2 (red line) and period 3 (green line) emerge at *δ* = 0.11 and *δ* = 0.31, respectively. Each of these undergoes period doubling as the forcing amplitude increases, eventually degenerating into chaos. A new period 4 cycle (blue line) exhibiting a period doubling cascade appears at *δ* = 0.60. As we shall see, each of these multi-annual attractors is the result of subharmonic resonance. Three important aspects of the model dynamics are worth emphasizing now: (i) the amplitudes of the new subharmonic oscillations are greater than that of the annual base mode; (ii) a subharmonic attractor may display period doubling as *δ* increases, eventually degenerating into chaos; and (iii) the model can be multi-stable when sufficient seasonality operates. These properties are qualitatively similar to the general features of seasonally forced SIR models (Dietz 1976; Aron & Schwartz 1984).

The genesis of subharmonic resonance cycles is best understood by examining response curves; this is the so-called resonance approach utilized by Greenman *et al*. (2004). These diagrams show the magnitude, *y*_max_, and the period, *p*_S_, of the system's response as a function of the external forcing period, *p.* The resultant diagram, which we refer to as a ‘resonance diagram’ for consistency with Greenman *et al*. (2004), explores the potential for resonance by revealing the relationship between the natural period of the system, *p*_0_, the external forcing period, *p*, and the period of the system's response, *p*_S_. It is important to realize that while annual forcing is usually the only relevant external period in an epidemiological context, the resonance diagram allows us to see how the system's responsiveness links to the natural and external forcing periods.

Figure 1*b* shows resonance diagrams for the baseline model at two different forcing amplitudes, corresponding to low (*δ* = 0.15, dotted line in figure 1*a*) and moderate (*δ* = 0.33, dashed line in figure 1*a*) forcing. The low and moderate amplitude cases exhibit a single attractor and a pair of coexisting attractors, respectively. Each attractor corresponds to an isolated structure exhibiting a peaked profile, which we characterize as either a ‘wave-like’ (e.g. the black–red–blue lines) or a ‘spray-like’ (e.g. the green line), by analogy with surface waves on water. These are colour coded according to the response period of the system (*p*_S_) relative to the external forcing period (*p*), such that a period 1 response (*p*_S_ = *p*) is black, a period 2 response (*p*_S_ = 2*p*) is red, a period 3 response (*p*_S_ = 3*p*) is green and a period 4 response (*p*_S_ = 4*p*) is blue. We refer to the *p*_S_ = *p* response region (i.e. black lines) as the ‘base peak’, because the system response is simply matching the external forcing period.

Under sufficient forcing, subharmonic resonance of nonlinear systems can occur when the external forcing period is near a submultiple of the natural period (i.e. *p*∼*p*_0_/*n* for *n* = 2, 3, 4, … ). These periods, easily calculated using linear stability analysis, are located by the vertical dotted lines in figure 1*b*. When the critical forcing amplitude is reached, a new period 2 subharmonic peak appears from the base peak by period doubling. We refer to the location on the forcing period axis where this new peak arises as the ‘root’ of the subharmonic. Period 2 subharmonic (*p*_S_ = 2*p*) should be rooted to the point *p*∼*p*_0_/2, and indeed, we see that the appropriate peak (red line) has emerged at this location under low forcing (*δ* = 0.15). As the forcing amplitude increases (*δ* = 0.33), we observe distortion of this peak to the right and further period doubling to create a period 4 (*p*_S_ = 4*p*) region. The base of the peak spreads out under increased forcing, such that the leftmost point at which period 2 peak is actually observed does not correspond exactly to its theoretical root at *p*∼*p*_0_/2; and the wave-like peak folds rightward over itself, generating an unstable attractor (dotted red line) and two coexisting stable attractors over the narrow folded region. Interestingly, this peak has a much wider base region that the corresponding peak for the measles example presented by Greenman *et al*. (2004). Simulations indicate that this difference is related to the long infectious period used in our model rather than the immune boosting feature. An important consequence of increased forcing is that higher order subharmonic peaks may appear. Thus, we observe the presence of period 3 peak when *δ* = 0.33. This spray-like peak is rooted at *p*∼*p*_0_/3, because if we project downwards from period 3 peak we arrive (approximately) at the point *p*_0_/3. In principle, distinct peaks corresponding to the fundamental mode and the series of harmonics rooted to the family of points *p*∼*np*_0_ (*n* = 1, 2, 3, … ) may also be expected. However, these overlap to such an extent that in fact a single base peak (*p*_S_ = *p*) plateau is formed towards the right of the diagram. As the focus of this study is subharmonic resonances, we do not consider this region further.

Resonance diagrams provide two key insights. First, they demonstrate that we can roughly locate potential subharmonic resonance attractors simply by calculating the natural period of the system, a property that depends only upon the intrinsic parameters. It is necessary, though not sufficient, for *p*_0_ < *n* in order for a period *n* subharmonic to be accessed by an annually forced system, because the resonance peaks are distorted rightwards. This condition is met when the corresponding resonance peak is rooted to the left of the line through *p* = 1. This rule is only an approximation because nonlinearities in the system result in spreading at the base of the peak. Nonetheless, it serves as a very useful rule of thumb for understanding when subharmonic resonance is possible. In our example, additional subharmonic peaks rooted to the family of points *p*∼*p*_0_/*n* (*n* = 4, 5, 6, … ) might be expected, and indeed period 4 attractor is observed in figure 1*a* when the forcing amplitude is sufficiently large. The second key reason for constructing resonance diagrams is that they provide insight into the ‘responsiveness’ of a system to periodic forcing. By making small changes to the parameters of a baseline case and examining changes in the response diagram, we can undertake a qualitative assessment of whether the system is more (or less) likely to exhibit resonance behaviour, by observing the genesis (or loss) of new subharmonic peaks and period doubling (or halving) of extant peaks. This assessment can be made whether or not the new behaviours are actually accessible under annual forcing.

## 4. Results

We now consider each key epidemiological parameter—the initial and boosted immune period, the infectious period and the vectoral capacity (i.e. transmission)—in turn to explore how they influence the potential for subharmonic resonance dynamics in malaria. In each case, a bifurcation diagram passing through the baseline case (parameters given figure 1) is generated, along with the resonance diagram resulting from a small perturbation to the baseline case. We combine insights from both diagrams to understand the general epidemiological conditions favouring subharmonic resonance. The standard bifurcation analysis allows us to describe the oscillatory behaviour of a system in great detail, but it can be very difficult to make general statements about the conditions favouring resonance behaviour. For example, a particular resonance mode may be absent because the natural period is too large (or small), because the forcing amplitude is too small to elicit resonance or because the forcing amplitude is large enough that the appropriate attractor has degenerated into chaos. This distinction is important, because in the latter two cases the observed dynamics are determined in part by the somewhat arbitrary choice of forcing function. Therefore, understanding the subtle interactions between the disease parameters and the strength of forcing, mediated through their effect on the natural period (*p*_0_) and the bifurcation structure, respectively, is difficult if we rely only on standard bifurcation diagrams. In contrast, comparing resonance diagrams at different points in the parameter space allows us to focus on how the epidemiological characteristics affect the propensity for resonance *per se*. In the following analysis, we adopt the moderate forcing example from figure 1 (*δ* = 0.33) as our baseline case.

### 4.1. Immune period

**Figure 2.**
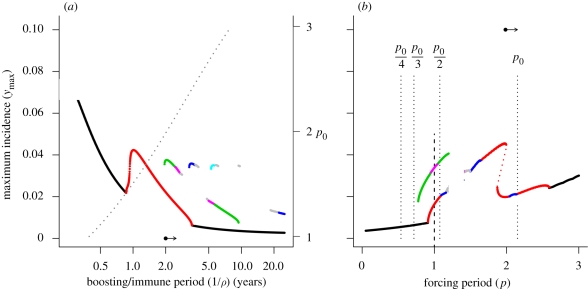
(*a*) Bifurcation diagram showing the resonant states of the model as a function of the immune period following recovery or immune boosting, 1/*ρ* (years). Remaining model parameters are the same as those given in figure 1. Arrow indicates the baseline parameter set (solid point) and the comparison case (arrowhead). Dotted line shows the natural period of the system (right vertical axis) as a function of the immune/boosting period. (*b*) Resonance diagrams for the comparison case, *δ* = 0.33 and *ρ* = 0.4 yr^−1^ (2.5-year immune/boosting period). Arrow indicates the natural period of this comparison case (arrowhead) relative to the baseline case (solid point). The vertical dotted lines locate submultiples of the natural period. Black line, *p*_S_ = 1; red line, *p*_S_ = 2; green line, *p*_S_ = 3; dark blue line, *p*_S_ = 4; light blue line, *p*_S_ = 5; pink line, *p*_S_ = 6; grey line, *p*_S_ ≥ 8.

Figure 2*a* depicts the bifurcation diagram for the initial and boosted immune periods (hereafter referred to as the immune period). Short periods of immunity, on the scale of weeks or months, are associated with purely annual patterns of variation. This is not surprising, given that the SIS model without acquired immunity is less likely to show resonance behaviour than a comparable SIR model. As the length of the immune period increases, a sequence of resonance cycles with increasing period is generated, in which successively higher period oscillations show an increased tendency to exhibit period doubling and degeneration to chaos. Figure 2*b* depicts the resonance diagram for a case in which the immune period (1/*ρ*) is increased relative to the baseline case, such that individuals remain immune for longer following recovery from infection or additional boosting of immunity (the arrow in figure 2*a* illustrates the parameter perturbation). Extending the time spent in the immune class clearly increases the tendency of the system to undergo subharmonic resonance; the extent of each resonance peak has increased and the period-doubling regions have widened relative to the baseline example (figure 1*b*, *δ* = 0.33), such that the period 2 peak now encompasses an extended period doubling cascade degenerating into chaos. Increasing immunity also increases the natural period of the system (illustrated by the arrow in figure 2*b*), shifting the root of each resonance peak to the right. Further extending the time spent in the immune class results in loss of access to successively higher period peaks as the natural period increases, so that eventually the system can only access the base peak and the period 4 peak. Importantly, the infected proportion of the population at the trough of a period 4 oscillation is very low (less than 10 infected individuals per 100 000 individuals), which means that stochasticity may disrupt subharmonic resonance at higher periods. Therefore, while extending immunity certainly increases the intrinsic tendency for subharmonic resonance, in practice, such states may not be attained because the natural period of the system is too high or because stochastic effects dominate. This argues that multi-annual cycles are most likely at intermediate levels of immunity (on the scale of a few years).

### 4.2. Infectious period

**Figure 3.**
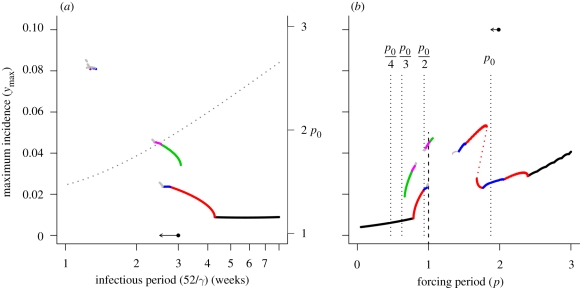
(*a*) Bifurcation diagram showing the resonant states of the model as a function of the length of the infectious period, 52/*γ* (weeks). Remaining model parameters are the same as those given in figure 1. Arrow indicates the baseline parameter set (solid point) and the comparison case (arrowhead). Dotted line shows the natural period of the system (right vertical axis) as a function of the infectious period. (*b*) Resonance diagrams for the comparison case, *δ* = 0.33 and *γ* = 20.8 yr^−1^ (2.5-week average infectious period). Arrow indicates the natural period of this comparison case (arrowhead) relative to the baseline case (solid point). The vertical dotted lines locate submultiples of the natural period. Black line, *p*_S_ = 1; red line, *p*_S_ = 2; green line, *p*_S_ = 3; blue line, *p*_S_ = 4; pink line, *p*_S_ = 6; grey line, *p*_S_ ≥ 8.

Figure 3*a* depicts the bifurcation diagram for the infectious period. Here multi-annual cycles are more likely to occur when the infectious period is shortened, although very short periods are associated with the complete loss of periodicity (and chaotic dynamics). Period 4 resonance is very unlikely to be attained under realistic conditions because stochastic effects will dominate at the cycle's trough. Figure 3*b* shows the resonance diagram for an instance in which the infectious period (1/*γ*) is reduced relative to the baseline case (the arrow in figure 3*a* illustrates the parameter perturbation). Reducing the infectious period has a similar effect on the tendency towards resonance as extending the time spent in the immune class. The base of both the period 2 and period 3 resonance peak has widened and the central region of each peak undergoes period doubling to chaos. However, despite the fact that resonance is more easily elicited relative to the baseline case (figure 1*b*, *δ* = 0.33), the system is only just able to access the period 2 and period 3 subharmonic resonances under annual forcing. In contrast to the previous example described in figure 2*b*, reducing the length of the infectious period also reduces the natural period of the system (illustrated by the arrow in figure 3*b*). If we continue to reduce the infectious period, access to the period 2 and period 3 subharmonics is lost because the line through *p* = 1 (annual forcing) crosses the chaotic region of each peak, rather than because the base of each peak is rooted to the right of this line. Access to the period 2 subharmonic can be regained by decreasing the forcing amplitude (results not shown), as this reduces the extent of the period doubling region. Finally, we observe relatively little change in the natural period as the infectious period decreases across the range of plausible infectious periods. Taken together, these results indicate that subharmonic resonance is promoted by lowering the infectious period, though these cycles may be destroyed if the forcing amplitude is too high.

### 4.3. Transmission

**Figure 4.**
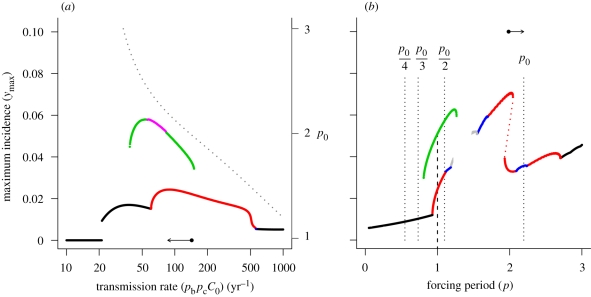
(*a*) Bifurcation diagram showing the resonant states of the model as a function of the transmission rate, *p*_b_*p*_d_*C*_0_ (yr^−1^). Remaining model parameters are the same as those given in figure 1. Arrow indicates the baseline parameter set (solid point) and the comparison case (arrowhead). Dotted line shows the natural period of the system (right vertical axis) as a function of the infectious period. (*b*) Resonance diagrams for the comparison case, *δ* = 0.33 and *p*_b_*p*_d_*C*_0_ = 86 yr^−1^. Arrow indicates the natural period of this comparison case (arrowhead) relative to the baseline case (solid point). The vertical dotted lines locate submultiples of the natural period. Black line, *p*_S_ = 1; red line, *p*_S_ = 2; green line, *p*_S_ = 3; blue line, *p*_S_ = 4; pink line, *p*_S_ = 6; grey line, *p*_S_ ≥ 8.

Figure 4*a* depicts the bifurcation diagram for the transmission rate (related to the mean vectorial capacity, *C*_0_). Here, multi-annual cycles are favoured at intermediate levels of transmission with seasonal outbreaks predicted at low and very high levels of transmission. Figure 4*b* shows the resonance diagram for an instance in which the vectorial capacity is reduced relative to the baseline case (the arrow in figure 4*a* illustrates the parameter perturbation), resulting in lower transmission. Reducing the magnitude of this term increases both the width of the period 2 and period 3 peaks and the extent of the period-doubling region inside the period 2 peak. Hence, the intrinsic likelihood of exciting subharmonic resonance increases as transmission is reduced. Some care must be taken when interpreting the effect of altering vectorial capacity, as this term concomitantly influences both the transmission of the disease and the effective length of the acquired immunity by means of its impact on boosting. Results presented in figure 2 indicate that reducing the length of the immune period decreases the intrinsic likelihood of subharmonic resonance and reduces the natural period of the system. However, the opposite response is seen when transmission is reduced; the natural period increases and the system is more responsive to forcing. Therefore, it appears that the direct effect of reduced transmission outweighs the indirect impact on immunity. We conclude that reducing transmission enhances the potential for subharmonic resonance, up to a point, after which resonance attractors become inaccessible because the natural period of the system is too high.

### 4.4. General results

In summary, the analysis presented above indicates that the likelihood of eliciting subharmonic resonance in malaria under periodic annual forcing is increased by: (i) extending the period individuals remain immune from the scale of months to years; (ii) shortening the length of the infectious period, possibly through medical interventions; and (iii) reducing the transmission rate in high transmission areas, for example by decreasing the vectorial capacity. While each of these processes increases the intrinsic likelihood of resonance, it is essential to consider the natural period of the system under study when trying to determine the likelihood of observing such behaviour. In the presence of annual forcing, subharmonic resonance at period *n* may only be observed if the (approximate) condition *p*_0_ < *n* is satisfied. Even when this simple criterion is met, the nature of the nonlinearities in the system and the forcing amplitude (*δ*) ultimately determine whether any such resonance is observed. For example, shortening the infectious period should always increase the likelihood of multi-annual cycles (or chaotic dynamics, if the forcing amplitude is sufficiently large) as it increases the intrinsic propensity towards resonance and concomitantly reduces the natural period of the system. In contrast, increasing the time individuals remain immune following infection increases the system's propensity towards resonance while simultaneously increasing the natural period. This implies that while there is always a minimum immune period for eliciting subharmonic resonance, there may also be a maximum immune period, above which the system is only able to access the annual seasonal attractor.

## 5. The effect of treatment

A major component of malaria epidemiology missing from the current model is that of intervention by drug treatment. We explored the potential impact of such treatment on the resonance behaviour of the model in the simplest manner possible, by including a term that moves individuals from the infected class (*y*) to the susceptible class (*x*) at a constant rate, ν. This gives rise to the following modified equations governing the dynamics of susceptible and infected populations:
5.1
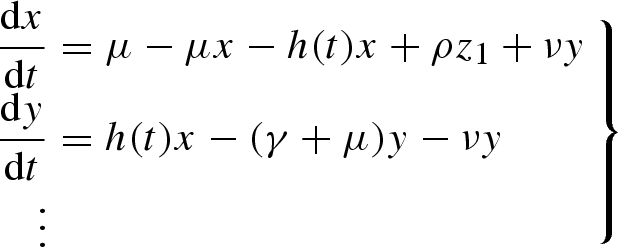



**Figure 5.**
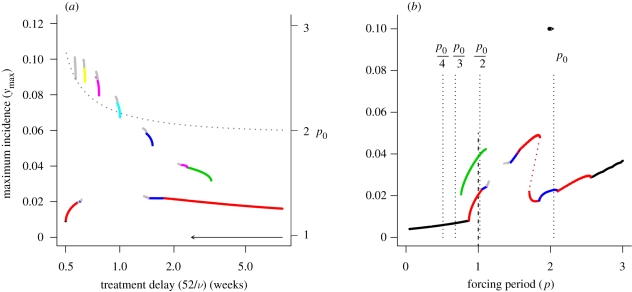
(*a*) Bifurcation diagram showing the resonant states of the model as a function of the time to treatment, 52/*ν* (weeks). Remaining model parameters are the same as those given in figure 1. Arrow indicates the baseline parameter set (solid point) and the comparison case (arrowhead). Dotted line shows the natural period of the system (right vertical axis) as a function of the infectious period. (*b*) Resonance diagrams for the comparison case, *δ* = 0.15 and *ν* = 20.8 yr^−1^ (2.5 weeks average time until treatment). Arrow indicates the natural period of this comparison case (arrowhead) relative to the baseline case (solid point). The vertical dotted lines locate submultiples of the natural period. Black line, *p*_S_ = 1; red line, *p*_S_ = 2; green line, *p*_S_ = 3; dark blue line, *p*_S_ = 4; light blue line, *p*_S_ = 5; pink line, *p*_S_ = 6; yellow line, *p*_S_ = 7; grey line, *p*_S_ ≥ 8.

Figure 5 shows a bifurcation diagram for the treatment parameter and the resonance diagram for the low treatment case. In contrast to the preceding examples, here we adopt the low forcing regime (*δ* = 0.15) from figure 1 as the baseline model. This is because much of the resonance structure is lost via degeneration into chaotic dynamics under the moderate forcing regime. Figure 5*a* depicts the bifurcation diagram for the time to treatment (referred to as the treatment delay). A sequence of resonance cycles with increasing period is generated as the treatment delay is reduced. Although the treatment model predicts that many modes of subharmonic resonance are possible under treatment, those above period 3 are very unlikely to be attained in reality. This is because disease incidence is very low following large epidemics (≪1 infected individual per 100 000 individuals), such that stochasticity will lead to local extinction and disruption of the resonance cycle. Figure 5*b* shows the resonance diagram for an instance in which the average treatment delay is 20 days. Even under the low forcing regime, the period 2 resonance peak has significantly broadened out and the usual period doubling cascade is present. In addition, a new period 3 resonance peak is visible (recall that the baseline case here is the *δ* = 0.15 example in figure 1). It is clear that including treatment significantly increases the likelihood of resonance behaviour under even very modest treatment regimes, presumably because it has a similar effect as reducing the infectious period in the treatment-free case. In contrast to simply reducing the infectious period, introducing treatment increases the natural period of the system. Moreover, the responsiveness of the system to forcing is much greater in the case of treatment.

## 6. Discussion

The simple model developed here offers some general insights into the conditions under which multi-annual (subharmonic resonance) cycles are likely to be observed in malaria. First, interventions that shorten the length of the infectious period, such as efficient treatment of infected individuals, will increase the likelihood of generating multi-annual cycles. Second, significant annual variation in transmission (i.e. variation of the order of at least 20–30%) is probably required in order to excite subharmonic resonance in the absence of treatment. This is a consequence of the fact that the infectious period of malaria is of the order of weeks rather than days, which in turn reduces the ease with which resonance is elicited. Third, cycles due to subharmonic resonance are most likely to occur in areas with low to moderate levels of disease transmission. High transmission reduces the likelihood of subharmonic resonance altogether, while under very low transmission the natural period of the system is too large for resonance to occur under annual forcing. We would predict that cycles are therefore most likely to be observed in meso-endemic (childhood infection prevalence, 10–50%; Metselaar & Van Theil 1959) regions.

We now return to the observation that malaria prevalence in the Kericho region of Kenya exhibits an apparent 3-year multi-annual cycle over the 1966–1998 interval (Hay *et al*. 2000). It has been suggested that malaria in much of the western highlands of Kenya is best classified as meso-endemic and seasonal (Hay *et al*. 2002). In the Kericho region, conditions are usually suitable for transmission throughout the year. This results in significant clinical immunity among adults, so that the disease is typically found in cohorts of children (Hay *et al*. 2001). In the period 1965–1998, the within-year range in the mean number of monthly malaria cases was approximately 6–20 in adults and 10–35 in children (Hay *et al*. 2001). Hence, two of our proposed requirements for subharmonic resonance in malaria, moderate year-round transmission and significant seasonal (annual) variation in transmission, are met in the region. The lack of evidence for a consistent 3-year cycle in the contemporaneous climate data for Kericho argues against the notion that such multi-annual variation in malaria is purely due to extrinsic drivers. Our model supports the proposition of Hay *et al*. (2000) that cycles in malaria prevalence in the region most likely result from an interaction between the extrinsic and intrinsic drivers of the disease. More precisely, we suggest that the observed cycles could occur as a result of subharmonic resonance of the system under annual forcing. Alternatively, it is possible that (non-seasonal) environmental variation and stochasticity lead to an alternative resonance phenomenon where the system oscillates at its natural period. A more recent analysis of malaria epidemics at two locations in the Kericho region used non-stationary time-series techniques (wavelets) and a fitted time-series SIR model to explore the role of climate variability and intrinsic factors in driving observed epidemic cycles (Pascual *et al*. 2008). Evidence for an approximate biennial cycle and a 3-year cycle in the data originally analysed by Hay *et al*. (2000) was reported, but the authors concluded that these were most likely driven by rainfall variation rather than intrinsic dynamics. The authors further concluded that a 4-year cycle was, however, likely to be driven by intrinsic factors at the second location, supporting the conclusions from our analysis that intrinsic drivers can lead to multi-annual cycles in this region. The time-series model that was fitted to the data assumed life-long immunity (Pascual *et al*. 2008) and as we have shown the nature of the immune response is a key determinant of resonance dynamics. Our work would therefore suggest that further analysis of the time series is required to determine the mechanisms that drive the dynamics in any particular location.

In contrast to the epidemics in Kenya, an analysis of malaria prevalence within 13 districts of the northern region of Thailand shows no evidence for the presence of multi-annual cycles over a 25-year period (Childs *et al*. 2006). Several of the provinces exhibit large variation in disease prevalence over the course of a year, indicating that they are subject to considerable annual variation in transmission. However, the remaining aspects of malaria epidemiology in Thailand are clearly quite different from the highlands of Kenya. Owing largely to the success of the long-established control programme, malaria is no longer considered a major health threat throughout most of Thailand. The annual parasite prevalence has recently been reported to be as low as 64 per 100 000 individuals (Thai Malaria Division 2003), and while the disease is still a problem in some forest fringe areas and along international borders (Somboon *et al*. 1998), it is best classified as hypo-endemic (childhood infection prevalence, ≪10%) throughout most of Thailand. Our model predicts that subharmonic resonance is extremely unlikely to occur under these circumstances, as the natural period of the system will simply be too high for any of the extant resonance peaks to be rooted to the left of the annual forcing case. Consequently, subharmonic resonance is unlikely to be observed, despite the fact that such behaviour is more easily excited under low transmission conditions.

The availability and quality of treatment obviously varies enormously throughout the world. In Thailand, effective diagnosis and treatment of the disease are freely available throughout the country as part of the ongoing control programme (Thai Malaria Division 2003). Kericho is unusual for much of sub-Saharan Africa in that the major employers in the region, the tea plantation owners, provide free malaria treatment for their workers (Shanks *et al*. 2005*b*). However, the situation is complicated by the fact that a large number of infections are contracted outside the region (Shanks *et al*. 2005*a*), raising the question as to whether the observed prevalence patterns truly reflect local processes. Because effective treatment is not widely available outside the Kericho region, this makes assessing the impact of treatment on the epidemiological dynamics very difficult. Clearly, a detailed study of the interaction between different forms of drug treatment and parasite life history is required to make general predictions about the ultimate impact of treatment on epidemiological dynamics.

Our model has made a number of broad predictions about the conditions favouring resonant behaviour in malaria dynamics. We predict that processes shortening the length of the infectious period very effectively promote resonance and that such behaviour is more likely to occur in regions with moderate seasonality in transmission and intermediate levels of transmission. We have made extensive use of the recently highlighted resonance approach to investigate the potential for subharmonic resonance in the epidemiological dynamics of malaria. This approach allows us to make statements about resonance behaviour *per se*, by removing our focus from the less general phenomenon of whether or not a particular resonance mode is observed under a particular annual forcing regime, with a given set of parameters. By varying each epidemiological parameter in turn, we are able to perform a form of local sensitivity analysis on the resonance behaviour of the baseline malaria model. As such, the approach is more instructive than simply generating bifurcation diagrams for each parameter in turn, though it clearly has limitations. In particular, the effect of altering multiple aspects of the epidemiology relative to the baseline case may be hard to predict, particularly in more complex models. Nonetheless, it would be valuable to explore the resonance behaviour of models with more biological details in order to determine the robustness of our predictions. In particular, the manner in which immunity is modelled is crucial to the dynamical outcomes and care should therefore be taken in assuming simple SIR dynamics in complex diseases such as malaria. For example, the degree to which the immune response is really maintained by boosting is still a matter of debate (Gupta *et al*. 1999; Struik & Riley 2004), and a model incorporating a graded immune response to repeated infection might offer a more realistic description of the disease. Related to this, many important parasites including malaria show substantial strain variation that is crucial to the infection dynamics (Gupta *et al*. 1998; Recker *et al*. 2004). Without doubt, there is a pressing need for better data to inform the more complex models that would be required to address these questions. However, the simple modelling framework developed here supports the parsimonious proposal that annual seasonality leads to the observed epidemics in Kenya and that the lower malaria prevalence in Thailand explains the lack of observed cycles despite a significant annual variation in transmission.
